# SERINC2-mediated serine metabolism promotes cervical cancer progression and drives T cell exhaustion

**DOI:** 10.7150/ijbs.105572

**Published:** 2025-01-20

**Authors:** Yixuan Sun, Yang Zhou, Qihua Peng, Wanzhen Zhou, Xiao Li, Ruiwen Wang, Yifan Yin, Huixian Huang, Hongfei Yao, Qing Li, Xueli Zhang, Lipeng Hu, Shuheng Jiang, Zhigang Zhang, Dongxue Li, Xiaolu Zhu, Yincheng Teng

**Affiliations:** 1 Department of Gynecology and Obstetrics, Shanghai Sixth People's Hospital Affiliated to Shanghai Jiao Tong University School of Medicine, Shanghai, 200233, P.R. China.; 2 State Key Laboratory of Systems Medicine for Cancer, Shanghai Cancer Institute, Ren Ji Hospital, School of Medicine, Shanghai Jiao Tong University, Shanghai, 200240, P.R. China.; 3 Department of Biliary-Pancreatic Surgery, Ren Ji Hospital, School of Medicine, Shanghai Jiao Tong University, Shanghai, 200127, P.R. China.; 4 Department of General Surgery, Pancreatobiliary Surgery Center, Huadong Hospital Affiliated to Fudan University, Shanghai, 200040, PR China.

**Keywords:** cervical cancer, SERINC2, tumor immune microenvironment, serine metabolism

## Abstract

Cervical cancer remains the most prevalent gynecological malignant disease. Reprogramming tumor immune metabolism stands out as a novel promising therapeutic target. Here, we identified serine incorporator 2 (SERINC2) as a critical gene which highly expressed in cervical cancer and negatively correlated with clinical outcomes. Through functional assays, SERINC2 was determined to play a pro-tumoral role both *in vivo* and *in vitro*. Besides, the growth of cervical cancer cells was found to be largely dependent on serine in a manner influenced by SERINC2. As a serine transport associated protein, SERINC2 knockdown significantly reduced cervical cancer cells' intracellular serine level and altered the serine-associated-lipid metabolism. Immune infiltration analysis revealed that SERINC2 was negatively associated with CD8^+^ T cell infiltration and function. More importantly, we demonstrated a competitive relation between cancer cells and immune cells brought about by SERINC2. Mechanistically, cancer cells SERINC2 preferentially competed for micro-environmental serine over CD8^+^ T cells and rendered T cell exhaustion. Overall, SERINC2 remodels cancer development and serine metabolism in the tumor immune microenvironment (TIME), establishing an immunosuppressive and pro-tumoral milieu.

## Introduction

Cervical cancer (cervical squamous cell carcinoma and endocervical adenocarcinoma, CESC) remains the most common gynecological malignant disease with high morbidity and mortality worldwide^1^. Despite being preventable, still, there is an urgent need for new approach targeting CESC. It is indicated that tumors remodel their metabolism program to meet the elevated requirement toward sugars, amino acids (AA) and lipids for supporting cell proliferation and metastasis^2^. Serine, as one of the non-essential AA, takes part in many biological processes. Serine is the precursor of glycine, cysteine and other phospholipids^3^. It is also the main carbon doner of serine, glycine and one carbon (SGOC) metabolism, supporting nucleotide synthesis and protein translation^4^, ^5^. Serine is so essential that both cancer cells and immune cells depended on it for proliferation or expansion^6^, ^7^. Serine has attracted growing attention while therapies targeting serine metabolic pathway and serine derived metabolites have presented new opportunities.

Indeed, tumor developed in concert with the adjacent cells including immune cells, fibroblasts, endothelial cells and biomolecules such as growth factors and chemokine. Together they constitute a hypoxia and sparse nutrient micro environment named tumor immune microenvironment (TIME)^8^-^10^. To benefit themselves, tumor cells sculpt the TIME as a pro-tumoral and immunosuppressive environment, where infiltrated with exhausted CD8^+^ T cells^11^-^13^. The exhaustion of T cells refers to the functionally impairment, sustained inhibitory receptor expression and metabolic dysregulation^14^, ^15^. The competition between tumor and immune cells is one of the reasons that triggered T cell exhaustion^16^. However, the inner mechanism is not fully revealed.

Serine incorporator 2 (SERINC2) belongs to the SERINC family comprised of five members, SERINC1-5^17^. The family proteins contain 10-11 transmembrane domains to facilitate incorporating serine into phosphatidylserine and sphingolipids^18^, ^19^. SERINC2 was reported to have a close correlation with tumor progression. A study of lung adenocarcinoma demonstrated that SERINC2 knockdown inhibited H1650 and A549 cells proliferation and migration via PI3K/AKT signaling pathway^20^. Besides, SERINC2 was found to predict an unfavorable prognosis of low-grade glioma^21^. Plus, it positively correlated with ovarian cancer and papillary thyroid carcinoma oncogenesis^22^,^23^. So far, the function of SERINC2 in cervical cancer remains unknown.

In this study, we investigated the functions of SERINC2 in cervical cancer through *in vitro* and *in vivo* experiments. Moreover, SERINC2 was found to play an important role in regulating serine metabolism and contribute to establish an immunosuppressive microenvironment via competing for serine in TIME.

## Methods

### Cell culture and transfection

HeLa, C-4i, SiHa, C-33A and ME-180 cells were preserved in State Key Laboratory of Systems Medicine for Cancer, Shanghai Cancer Institute, Ren Ji Hospital, School of Medicine, Shanghai Jiao Tong University. The cell lines were authenticated by short tandem repeat (STR) profiling. All CESC cells were maintained in Dulbecco's modified Eagle's medium (DMEM, GIBCO, Cat#11995065, USA) supplemented with 10% fetal bovine serum (FBS, VivaCell, Cat#C04001-500, Shanghai) and 1% penicillin/streptomycin (P/S, GIBCO, Cat# 15070063). All cells were incubated at 37°C with 5% CO_2_.

siRNA and shRNA targeting against SERINC2 were purchased from Gene Pharma (Shanghai, China) and the sequence were as si*SERINC2*-1: 5'- GCAUUGUGGGCCUCAUCAUTT-3', si*SERINC2*-2: 5'-CCAACAUCUGGUUCUACUUTT-3'. Sequence of shSERINC2 ultimately applied in the *in vivo* experiment was 5'-CCGGATCACCCTCTACACCATGTTTCTCGAGAAACATGGTGTAGAGGGTGATTTTTTTG-3'. SERINC2 overexpression was achieved using GV705-SERINC2-FLAG synthesized by Genechem (Shanghai, China). Stable cell line was maintained in the complete medium with 7μg/mL of puromycin. Transient transfection was performed using jetPRIME ® (Polyplus, France, Cat#101000046) according to the manufacturer's instructions.

### Human CESC samples and tissue microarray

All CESC samples and tissue microarray were obtained from Department of Pathology, Shanghai Sixth People's Hospital Affiliated to Shanghai Jiao Tong University School of Medicine under the approval of Ethical Review Board. Patients' medical information was collected and analyzed from Department of Gynecology, Shanghai Sixth People's Hospital. Immunohistochemistry (IHC) staining was performed as previously described^24^. For SERINC2 IHC quantification, each tumor slide was assigned with a score achieved by staining intensity (0=negative, 1=weak, 2=moderate, 3=positive) multiplied by staining area (0=0%, 1=1-10%, 2=11-50%, 3=51-80%, 4=81-100%). The proportion of Caspase 3 and TUNEL-positive cells was determined by counting total number of immunostaining positive nuclei in the field. CD8, GZMB and LAG3 quantification were calculated based on pixel intensity. The quantification was performed by two pathologists in a blinded manner.

### CESC cell functional assay

Cell Counting Kit-8 (CCK-8) (KTA1020, Abbkine, China, Cat# BMU106) was applied for the cell viability assay. CESC cells were seeded in 96-well plate at the density of 2х10^3^ cells/well with quintuplicate technical replication. Cells were incubated at 37°C for 1 hour after adding CCK-8 reagent. Cell viability was measured under the absorbance wavelength of 450nm by microplate reader (M1000 PRO, TECAN). The experiments were performed in triplicates. 2х10^3^ cells were seeded into 6-well plate for cell colony formation assay. Cells were fixed by paraformaldehyde after 10-14 days followed by crystal violet staining and counting macroscopically. All experiments were performed in triplicates. For cell migration assay, the cervical cancer cells were pre-treated and loaded in the upper chamber of well (Falcon, USA, Cat#353097). The cells were resuspended in FBS free DMEM. After 24 hours, chambers were rinsed and fixed by paraformaldehyde. The chambers were subjected to crystal violet staining, while cells at the inner side of the chamber were wiped away. The bottom of the chamber was imaged by microscope.

### Quantitative real time PCR (qPCR) and Immunoblotting

Total RNA extraction and RNA reverse transcription were performed by applying SteadyPure Quick RNA Extraction Kit and Evo M-MLV RT Mix Kit (ACCURATE BIOLOGY, China, Cat#AG21023, Cat#AG11728). SYBR green Premix Pro taq HS qPCR Kit (ACCURATE BIOLOGY, China, Cat#AG11701) were used to realize PCR at the recommended thermal setting. Primer sequences: *SERINC2* (human) forward: 5'-CCTTCTGTGTCTGCGTGTCCATC-3', reverse: 5'-GATAGGGCTGACCAGGTGACAAAC-3', *18s* (human) forward: 5'-GGCCCTGTAATTGGAATGAGTC-3', reverse: 5'-CCAAGATCCAACTACGAGCTT-3'. All primers were synthesized by Sangon Biotech (China).

The cell membrane and cytosol protein extraction assay were described in supplementary file. Immunoblotting was performed as previously described^25^. Antibodies used were rabbit-anti-SERINC2(1:1000, SIGMA, Cat#A116103, USA), mouse-anti-β-actin (1:3000, Servicebio, Cat#GB12001, China), GOAT Anti-Rabbit IgG (H+L) HRP (1:5000, Abways, Cat#AB0101, China), Goat Anti-Mouse IgG (H+L) HRP (1:5000, Abways, Cat#AB0102, China).

### Cell immunofluorescence assay

HeLa and C-4I cells were plated in the 8 well chamber slide (ibidi, Germany, Cat#80826) and fixed by 4% paraformaldehyde for 30 mins at room temperature, followed by 10% (m/v) bovine serum albumin (BSA, Biofroxx, China, Cat#9048-46-8) blocking and a secondary antibody (Servicebio, China, Cat# GB28301, Abbkine, China, Cat#A22110) incubation for 1 h at room temperature. Then the chamber slide was counterstained with DAPI (Servicebio, China, Cat#GDP1024) and ready for visualization.5-Ethynyl-2′-deoxyuridine (EdU) assay detection kit (Beyotime Biotechnology, China, Cat#C0071S) was used according to manufacturer's instruction. 3х10^4^ cells were plated in the 8 well chamber slide incubated with EdU (50μmol/l) for 2h at 37°C, followed by paraformaldehyde fixation and 0.5% Triton X-100 permeabilization. Cells were then stained with Click Additive Solution and DAPI for visualization. JC-1 mitochondrial membrane potential assay kit (ShareBio, China, Cat#SB-J6004) was applied following manufacturer's protocol. 3х10^4^ cells were incubated with JC-1 working buffer at 37°C for 15 mins. Cells were rinsed and imaged. All samples were visualized by Zeiss LSM 510 (Zeiss, Germany).

### Xenograft assays

All experimental animals were maintained at specific pathogen free (SPF) Laboratory Animal Center according to the guidelines by the NIH Guide for laboratory animals care and application. The experimental protocols were approved by animal ethics committee of the Shanghai Sixth People's Hospital Affiliated to Shanghai Jiao Tong University School of Medicine. For xenograft experiment, 5-week-old BALB/C nude female mice were purchased from Shanghai SLAC Laboratory. Animals were allowed to familiarized the new environment for 1 week. Then, 2 х 10^6^ HeLa cells stably expressing shNC/shSERINC2 were injected subcutaneously on the right/left posterior axillary line of each mouse. Tumors were measured every week. 4 weeks after the injection, all mice were sacrificed and the tumors were harvested. Tumor weight and volume were recorded and analyzed. Then, all tumor samples were subjected to IHC staining.

C-NKG (NOD-*Prkdc^scid^ Il2rg^em1/Cyagen^*) mice were purchased from Cyagen (China). For C-NKG mice immune reestablishment, 5х10^6^ human peripheral blood monocyte cells (JUNXBIO, China) were injected intraperitoneally. At day 5, CD45 (PE anti-human CD45 Antibody, Biolegend, Cat#304007, PerCP/Cyanine5.5 anti-mouse CD45 Recombinant Antibody, Biolegend, Cat#157611) were detected using mice peripheral blood to determine the establishing efficacy. Human CD45 positive rate/(human CD45 + mouse CD45) positive rate>25% was deemed as efficacious. After 1 week, 4 х 10^6^ shNC/shSERINC2 HeLa cells were injected subcutaneously and simultaneously feed two group of mice with serine- and glycine- free diet. The other two group of mice continued with normal diet. Mice were sacrificed 2 weeks after the injection and tumors were harvested subjected to IHC staining.

### CD8^+^ T cells purification and functional assay

Blood samples were obtained from healthy donors with written informed consent under the approval of Ethical Review Board. The isolation of peripheral blood mononuclear cells (PBMC) was performed by standard density-gradient centrifugation protocols using Ficoll-Paque^TM^PLUS (cytiva, Sweden, Cat#17144002). Then CD8^+^ T cells were positively selected by magnetic CD8^+^ microbeads (Miltenyi Biotec, Germany, Cat#130-045-201)^26^. CD8^+^ T cells were maintained in TexMACS^TM^ GMP Medium (Miltenyi Biotec, Germany, Cat#7211000047) containing 5μg/ml of anti-human CD28 (BioLegend, USA, Cat# 302934) and 40ng/mL of IL2 (Biolegend, USA, Cat# 791904) in a plate previously incubated with 5ug/ml of anti-human CD3 (BioLegend, USA, Cat# 317326). After 48h of activation, CD8^+^ T cells were co-cultured with CESC cells for 24-48h. For CD8^+^ T cells functional assay, T cells were then collected and resuspended in buffer contained Phorbol 12-myristate 13-acetate 200ng/mL (PMA, Beyotime, China, Cat#S1819), ionomycin 300ng/mL (Beyotime, China, Cat#S1672) and Brefeldin A 1μg/mL (Macklin, China, Cat#B802056). Mixed the cell resuspension mixture well every hour. 4 hours later, CD8^+^ T cells were stained with anti-human GZMB (BioLegend, USA, Cat# 372213), anti-human TNF-α (BioLegend, USA, Cat#502973), anti-human PD-1 (BioLegend, USA, Cat# 329931) and anti-human CTLA-4 (BioLegend, USA, Cat#349927). All experiments were performed in triplicates.

### Metabolomics

Cells were collected and resuspended in cold 80% methanol, followed by 5 times freeze-thaw cycles to be fully lysed. Then the samples were subjected to centrifugation with 15000g at 4°C. The supernatant was dried under gentle nitrogen and reconstituted in 10% aqueous methanol. For non-targeting metabolomics analysis, the samples were submitted to Shanghai Xu Ran Biological Company. The chromatographic separation was performed on a ThermoFisher Ultimate 3000 UHPLC system with a Waters ACQUITY UPLC BEH C18 column (2.1mm × 100 mm, 1.7μm). The eluents were then analyzed on a ThermoFisher Q Exactive™ Hybrid Quadrupole-Orbitrap™ Mass Spectrometry (QE). The raw data were preprocessed by Compound Discoverer (verison 3.3, Thermo Fisher).

For 1-13C L-Serine detection, the C-4I cells were co-cultured with CD8^+^ T cells for 4h in serine/glycine free medium after 48h activation of CD8^+^ T cells. The medium was additionally added 10mg/L of 13C L-serine (Cambridge Isotope Laboratories, USA, Cat#CLM-1573-0.25). Then, C-4I, the supernatant and CD8^+^ T cells were prepared individually as mentioned above and submitted to Wuhan Servicebio Technology. For lipid-targeted metabolomic study, siNC/si*SERINC2* HeLa cells were prepared as previously mentioned and submitted to Shanghai Xinying Biotech Company.

### Bioinformatic analysis

The RNA sequencing data of CESC patients were downloaded using The Cancer Genome Atlas (TCGA) biolink. RNAseq data from normal cervical tissue were downloaded from Genotype Tissue Expression (GTEx) database. Then, the RNAseq count data were combined and grouped as tumor (n=306) and normal tissue (n=13). DESeq 2 was applied to detect differentially expressed genes (DEGs). Fold change (FC) ≥0.5, *P*<0.05 were set as restriction to determine DEGs. DEGs were verified in Gene Expression Omnibus (GEO) datasets. Serine biosynthesis and transport gene sets were obtained from Gene Set Enrichment Analysis (GSEA). Prognosis analysis was performed on Gene Expression Profiling Interactive Analysis (GEPIA) and Kaplan-Meier plotter (KM plotter). Immune cells infiltration score was analyzed by CIBERSORT algorithm according to SERINC2 mRNA expression^27^. Single cell RNA sequencing database was analyzed by Tumor Immune Single-cell Hub (TISCH). Prognosis analysis of CD8^+^ T cell infiltration and SERINC2 mRNA expression was applied by TIMER2.0^28^-^30^. The association between the gene expression and immune infiltrates was performed by Spearman correlation analysis.

### Statistical analysis

All statistical analysis was performed by IBM SPSS Statistics (Version 25.0) and graphs generation and representation were completed by GraphPad Prism 8 (San Diego, USA). All data in histogram was represented as mean ± standard deviation (SD) and the significance was calculated by Student's t-test or one-way ANOVA.* P* < 0.05 was considered statistically significant.

## Results

### Identification of SERINC2 as a critical serine associated gene in CESC development

**Figure 1:**
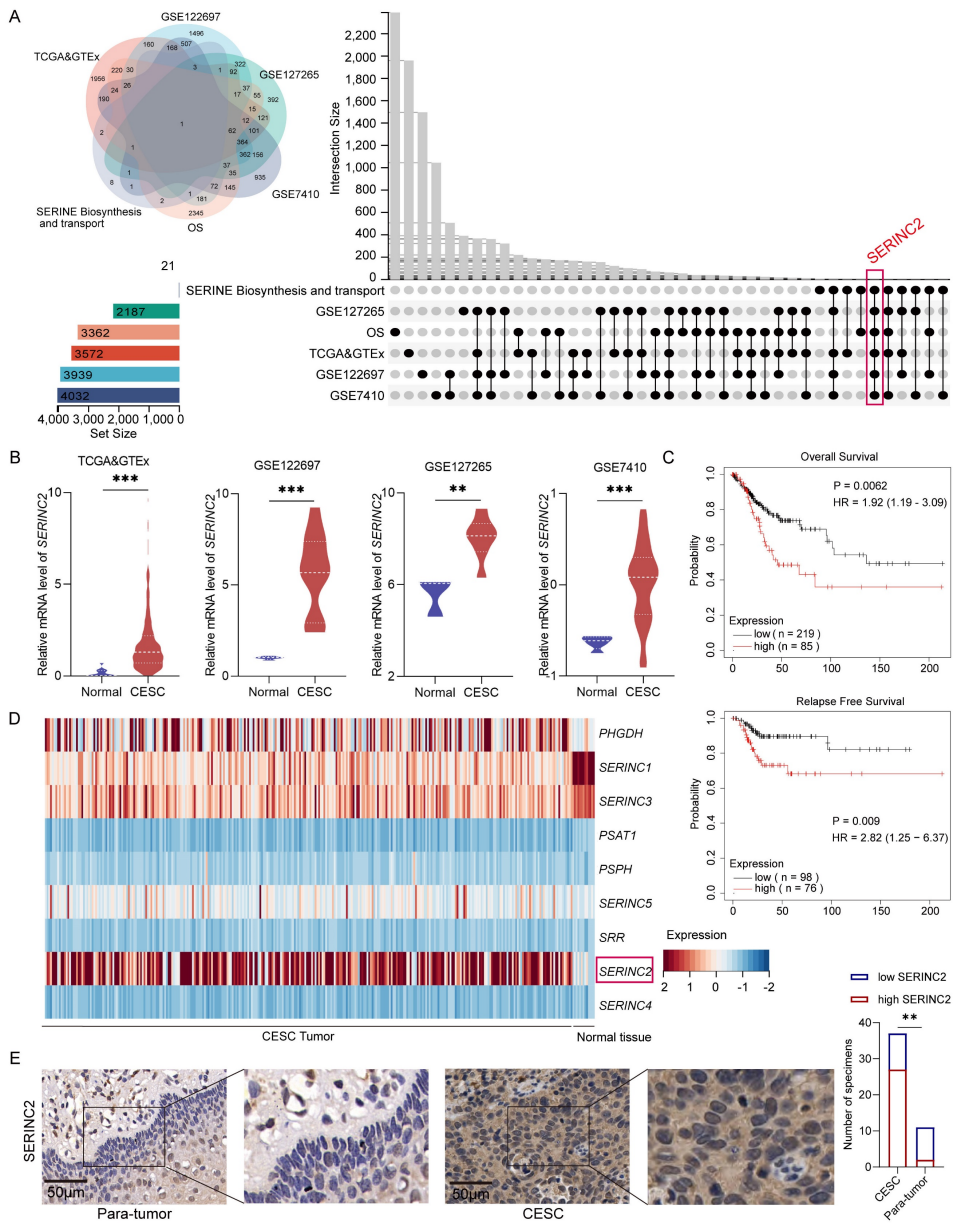
Identification of SERINC2 as a critical serine associated gene in CESC development. **(A)** Identification of SERINC2 in TCGA, GTEx, GSE122697, GSE127265, GSE7410 and GSEA database using Venn diagram. **(B)** Expression profile of SERINC2 in TCGA&GTEx, GSE122697, GSE127265 and GSE7410 database. **(C)** Kaplan-Meier analysis of overall survival and relapse free survival in patients with high or low SERINC2 expression using GEPIA and KM plotter online server. **(D)** Heatmap of serine biosynthesis and transport gene set in GSEA using TCGA RNAseq expression profile. **(E)** Representation of IHC staining of SERINC2 expression in 37 cases of cervical cancer/adjacent tissue sections and statistical analysis. Scale bar=50μm. *P < 0.05, **P < 0.01, ***P < 0.001.

To identify the key serine associated gene in CESC development, we initially mined the CESC RNAseq expression files in TCGA and GTEx database to determine the upregulated DEGs in tumor, which was 3572 genes in total. Then the upregulated DEGs were further verified by analyzing GEO database GSE122697, GSE127265 and GSE7410 to screen the overlapped genes. Next, to sort out those genes with significant poor prognosis in CESC patients, we employed online website GEPIA^31^ and KM plotter^32^ to narrow the chosen genes. More importantly, we explored serine biosynthesis and transport gene sets on GSEA^33^, ^34^, where we ultimately focused on *SERINC2* (Fig. 1A)*.* As a result, *SERINC2* mRNA expression was significantly higher in tumor compared to normal tissue (Fig. 1B). In addition to that, SERINC2 was negatively correlated with overall survival (OS) and relapse free survival (RFS) in CESC (Fig. 1C). Besides, we have noticed that the other SERINC family genes were also included in the GSEA gene sets (Fig. 1D). Then we analyzed their expression in TCGA and GTEx database (Supplementary Fig. 1A). Despite the fact that *SERINC5* showed higher expression in tumor as well, it did not determine the prognosis of CESC patients (Supplementary Fig. 1B). Subsequently, we detected the expression of SERINC2 in tumor samples from cervical cancer patients and confirmed the upregulation of SERINC2 protein expression in tumor (Fig. 1E). Collectively, SERINC2 showed higher expression in cervical cancer and correlated with poor prognosis, suggesting its importance in CESC development.

### The proliferation of CESC cells requires serine and it is SERINC2-dependent

**Figure 2:**
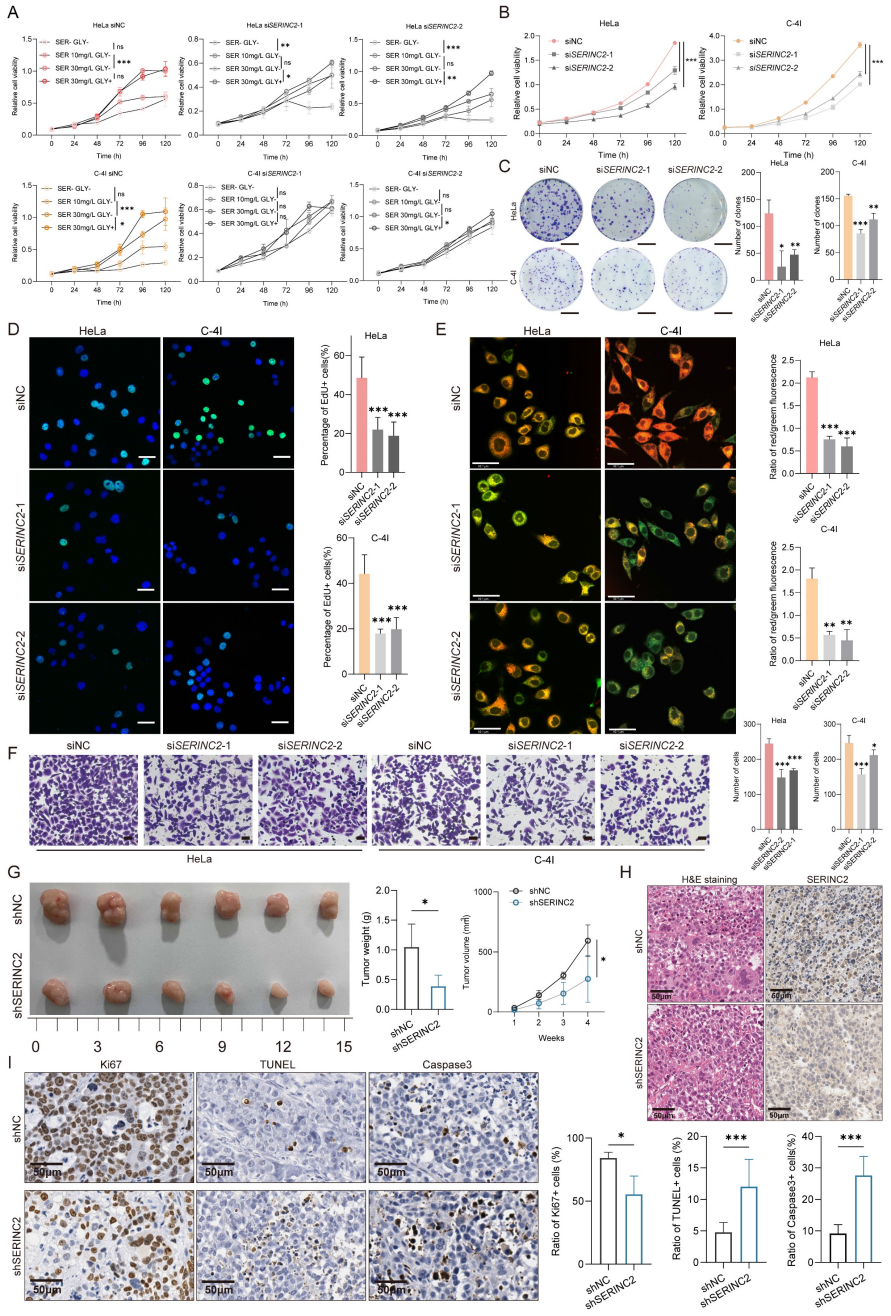
SERINC2 promotes cervical cancer tumorigenesis *in vitro* and *in vivo*. **(A)** CCK-8 assay analysis of HeLa and C-4I cell line under different serine concentration (serine 0mg/L, 10mg/L, 30mg/L, without glycine and serine 30mg/L with glycine 10mg/L). **(B)** CCK-8 analysis of HeLa and C-4I cell viability after SERINC2 downregulation. **(C)** Representation of colony formation assay and its analysis of SERINC2 knockdown Hela and C-4I cells. Scale bar=1 cm** (D)** Representative EdU staining and quantification analysis in SERINC2 knockdown HeLa and C-4I cells. Scale bar = 50μm.**(E)** Representative JC-1 staining images and quantification analysis in SERINC2 knockdown HeLa and C-4I cells. Scale bar = 50μm.** (F)** Representative images of transwell assay and its analysis in SERINC2 knockdown HeLa and C-4I cells. Scale bar=100μm. **(G)** Tumorigenesis of shNC/shSERINC2 HeLa cells in BALB/C nude mice, tumor growth curve and weight comparison of terminal tumors. Scale bar=1 cm. **(H)** H&E staining and SERINC2 IHC staining of subcutaneous tumor sections. **(I)** IHC staining of Ki67, TUNEL and Caspase3 for subcutaneous tumor sections from nude mice and statistical analysis, scale bar=50μm.*P < 0.05, **P < 0.01, ***P < 0.001.

To gain a deeper insight into the role of SERINC2 in cervical cancer progression, we firstly detected the mRNA and protein expression of SERINC2 in the cervical cancer cell line that preserved in our laboratory (Supplementary Fig. 3A). Given the association of SERINC2 with serine metabolism, we aimed to investigate the necessity of serine for the growth of cervical cancer cells. To this end, we chose HeLa, C-4I, SiHa and C-33A cell line, which expressed higher level of SERINC2, for the following experiments. These cell lines were cultured in medium with varying serine concentrations, ranging from serine/glycine free,10/30mg/L of serine with glycine free to 30mg/L of serine with 10mg/L of glycine. Glycine was also removed as it can buffer serine via one carbon metabolism. The CCK-8 assay showed that HeLa, C-4I, SiHa and C-33A cell line depended on serine for cell growth, particularly evident in HeLa and C-4I cells (Supplementary Fig. 2). We then downregulated SERINC2 in HeLa and C-4I with small interfering RNA (Supplementary Fig. 3B). Remarkably, knockdown of SERINC2 attenuated cells' dependency on serine for proliferation (Fig. 2A). In summary, the growth of cervical cancer cell lines relies on serine, and this dependency is mediated by SERINC2.

### SERINC2 promotes the tumorigenesis and progression *in vitro* and *in vivo*

To further investigate the function of SERINC2 in cervical cancer, we applied siNC/si*SERINC2* HeLa and C-4I cells for the subsequent experiments. It was showed that the knockdown of SERINC2 significantly impaired cell viability and proliferation in normal culture medium (Fig. 2B-C). EdU staining also corroborated these results (Fig. 2D, Supplementary Fig. 3F), indicating a crucial role for SERINC2 in CESC cell growth. Furthermore, SERINC2 down-expression led to increased cells apoptosis (Fig. 2E, Supplementary Fig. 3G). Here we applied JC-1 staining assay to substitute Annexin V as SERINC2 was reported to participate in phosphatidylserine biosynthesis, which functioned as the binding target of Annexin V (Supplementary Fig. 3E). In addition, we also assessed the impact of SERINC2 on cell migration. As a result, downregulation of SERINC2 significantly weakened HeLa and C-4I cell migration ability (Fig. 2F). Collectively, these findings suggest that SERINC2 systematically promotes cervical cancer tumorigenesis. Next, we established SERINC2 stably knockdown HeLa cells using lentivirus and validated the knockdown efficiency (Supplementary Fig. 3D). The shNC/shSERINC2 HeLa cells were injected subcutaneously into BALB/C nude mice. Expectedly, SERINC2 knockdown impaired cell growth *in vivo*, evidenced by significantly reduced tumor volume and weight (Fig. 2G). Concurrent IHC staining demonstrated that SERINC2 promoted cell proliferation and enhanced resistance to apoptosis and cell death (Fig. 2H-I). Taken together, SERINC2 plays a pro-tumor role *in vitro* and *in vivo*.

### SERINC2 locates to cell membrane and modulates intracellular serine level

**Figure 3:**
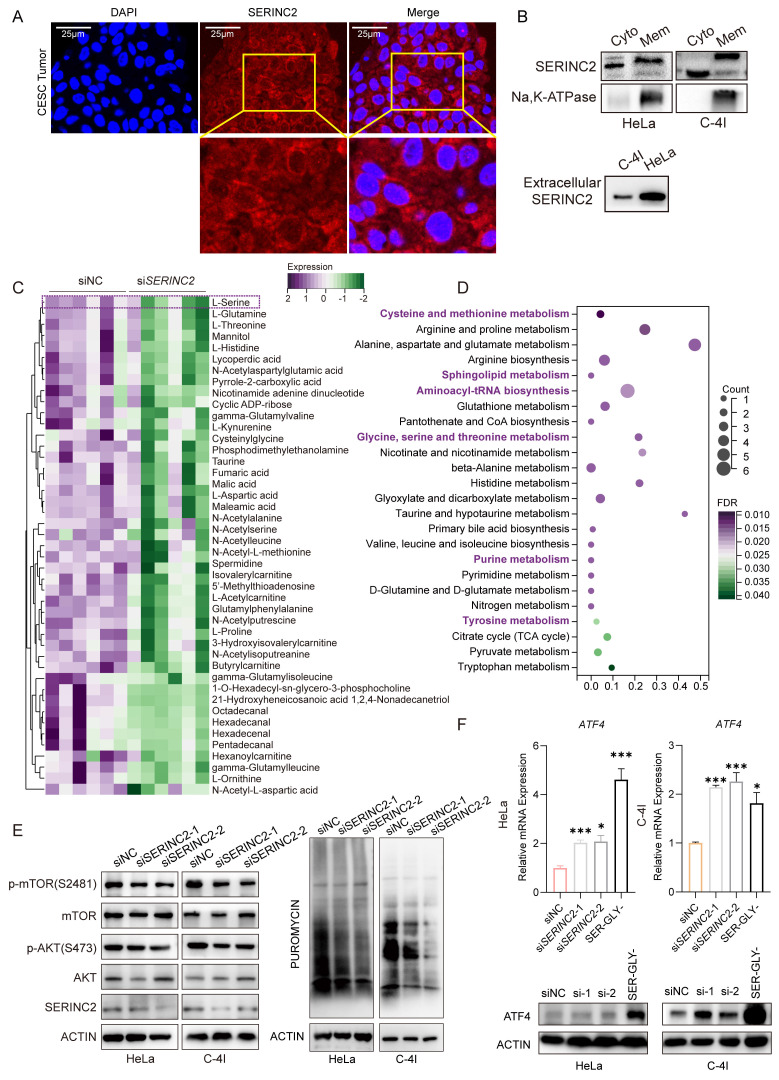
SERINC2 locates to cell membrane and influences intracellular serine level. **(A)** Immunofluorescence staining to detect SERINC2 localization in CESC patients' tumor samples, scale bar= 25 μm. **(B)** Immunoblotting using protein extracts from cytoplasm, membrane and cell supernatant to determine SERINC2 expression HeLa and C-4I. Cyto: cytosol, Mem: cell membrane. **(C)** Differential downregulating metabolites in Hela siSERINC2 compared to siNC cells using LC-MS. **(D)** Pathway enrichment analysis of differential metabolites. **(E)** Western blotting was applied to detect change in the AKT-mTOR pathway after SERINC2 knockdown. Puromycin infiltration assay was used to detect the synthesis of nascent protein after SERINC2 knockdown.** (F)** mRNA protein level expression of ATF in SERINC2 knockdown HeLa, C-4I cells under normal complete medium conditions and serine/glycine deprived cultured HeLa and C-4I cells. The serine withdrawal time was 24h. *P < 0.05, **P < 0.01, ***P < 0.001.

Our subsequent inquiry focused on the precise subcellular localization of SERINC2 and its impact. We detected the expression of SERINC2 in human tumor samples as well as cell lines and confirmed its presence in both cell membrane and cytoplasm, as anticipated (Fig. 3A, Supplementary Fig. 4A). Subsequently, we isolated protein from cell membrane and cytosol for immunoblotting. It was found that the membrane-bound SERINC2 exhibited a larger molecular mass compared to cytoplasmic SERINC2 (Fig. 3B, Supplementary Fig. 4B), indicating that SERINC2 probably exert its function through post-translational modification. In addition, protein extracted from the cell supernatant confirmed the presence of SERINC2 in the extracellular milieu (Fig. 3B). Given SERINC2's predominant localization at the cell membrane, we hypothesized that it probably involved in serine uptake. To this end, siNC/si*SERINC2* HeLa cells were subjected to a non-targeting metabolomics analysis. As a result, SERINC2 downregulation significantly decreased intracellular serine level of HeLa cells (Fig. 3C). Moreover, the KEGG pathway analysis demonstrated that SERINC2 knockdown influenced serine and serine-derived metabolism pathway (Fig. 3D).

Based on the aforementioned findings, SERINC2 regulates intracellular serine levels, prompting us to investigate the extent of this modulation. Hence, we examined two major AA sensing signaling pathway, ATF4 and mTOR pathways^35^, ^36^. We also conducted a puromycin incorporation assay to assess whether protein translation was hindered due to amino acid intake limitation following SERINC2 downregulation. Notably, under conditions of ample nutrients in complete medium, downregulation of SERINC2 already diminished phosphorylation level of mTOR and AKT. Besides, SERINC2 knockdown also reduced synthesis of nascent protein (Fig. 3E). As was reported, AA deprivation increased uncharged tRNAs and thus activated GCN2-ATF4 pathway. In according with our expectations, we found down-expression of SERINC2 elevated *ATF4* mRNA expression significantly, albeit resulting in a modest increase in protein expression (Fig. 3F).

As serine involved in SGOC metabolism pathway, we then aimed to explore the influence of SERINC2 on SGOC metabolites. However, the non-targeting metabolomics analysis showed no difference between HeLa siNC and si*SERINC2* in term of all the SGOC downstream metabolites (Supplementary Fig. 4C). To further explore whether SERINC2 played its function in cytoplasm, we detected mRNA level of SGOC metabolism enzymes in siNC/si*SERINC2* HeLa and C-4I cells. Unlike to our expectation, only few enzymes' RNA level has decreased such as the folate metabolism enzymes *TYMS* and *DHFR* (Supplementary Fig. 4D). Since folate metabolism is critical for purine and thymidine monophosphate biosynthesis, we thus tested the intracellular level of ATP and GTP level. Both kit assay and LC-MS showed no difference between siNC and si*SERINC2* (Supplementary Fig. 4E-F). In conclusion, while SERINC2 deficiency leads to decreased intracellular serine levels, its role does not extend to the SGOC pathway.

### SERINC2 suppresses CD8^+^ T cells infiltration in CESC

**Figure 4:**
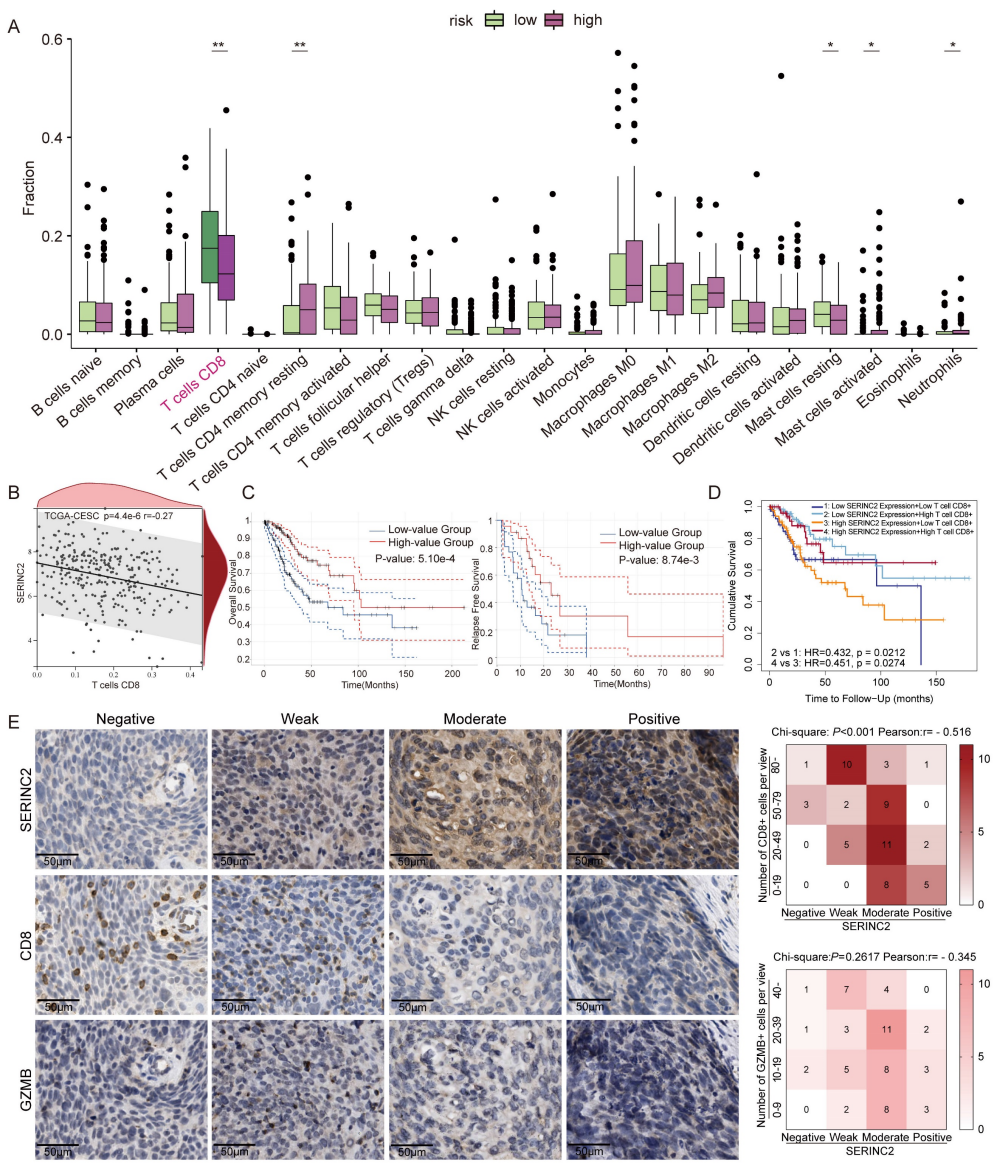
SERINC2 participates in regulating CESC tumor immune microenvironment. **(A)** CIBERSORT analysis of the correlation between SERINC2 mRNA expression level and immune cells infiltration. **(B)** Correlation analysis between CD8^+^ T cells and SERINC2. **(C)** Prognosis analysis of immune infiltration of CD8^+^ T cells with OS and RFS in the TCGA CESC database using GEPIA. **(D)** Prognosis analysis of CD8^+^ T cell infiltration and SERINC2 mRNA expression level with OS in the TCGA CESC database using TIMER2.0.** (E)** IHC staining of SERINC2, CD8 and GZMB in clinical cervical cancer samples and correlation analysis, scale bar=50μm. *P < 0.05, **P < 0.01, ***P < 0.001.

As SERINC2 regulated intracellular serine level and located to cell membrane, we hypothesized its significance in the TIME. To validate this conjecture, we performed immune infiltration analysis using TCGA database. Intriguingly, a negative correlation was observed between high SERINC2 expression in CESC patients and CD8^+^ T cell infiltration (Fig. 4A-B). Furthermore, online database analysis revealed a positive correlation between CD8^+^ T cells infiltration and overall survival in CESC patients (Fig. 4C). By incorporating both SERINC2 expression and CD8^+^ T cell infiltration data, we observed that patients with high SERINC2 expression and low CD8^+^ T cell infiltration had the poorest clinical outcomes (Fig. 4D). This rendered us to speculate that SERINC2 contributed to the establishment of an immune-suppressive environment via inhibiting CD8^+^ T cells infiltration and its cytotoxic ability. To this end, IHC staining of consecutive slides of tumor samples from CESC patients were conducted, it confirmed a negative association between SERINC2 expression and CD8^+^ T cell infiltration at the protein level (Fig. 4E). Besides, we also observed a negative correlation trend between expression of SERINC2 and Granzyme B (GZMB), a cell-death-targeting molecular secreted by cytotoxic T cells. Collectively, SERINC2 inhibits CD8^+^ T cells infiltration to facilitate remodeling an immunosuppressive and tumor-promotive microenvironment.

### SERINC2 competes for serine in TIME and drives CD8^+^ T cell exhaustion

**Figure 5:**
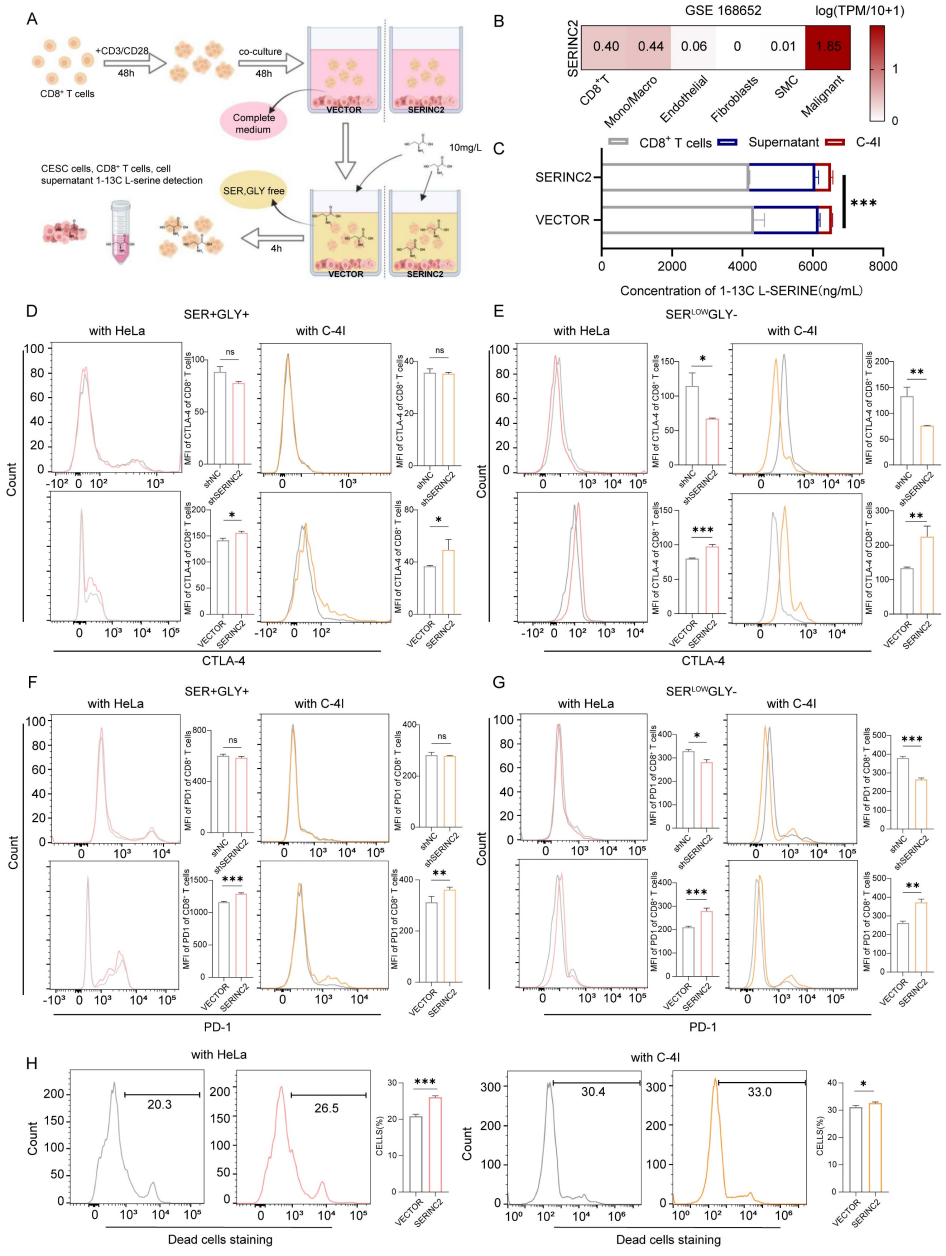
SERINC2 competes for serine in tumor immune microenvironment and drives CD8^+^ T cell exhaustion. **(A)** Diagram of samples preparing procedure before L-serine detection.** (B)** Analysis of single-cell RNA sequencing of cervical cancer tissues and normal adjacent tissues by TISCH in GSE168652 database.** (C)** LC-MS determination of L-serine-1-13C concentration in C-4I cells, co-culture supernatant and CD8^+^ T cells, the cell number ratio of immune cells versus tumor cells was 1 to 5, VECTOR: Control group, SERINC2: SERINC2 overexpression. **(D-E)** Detection of CTLA-4 expression level in CD8^+^ T cells co-cultured with SERINC2 overexpressed/knockdown HeLa/C-4I cell lines in SER+/GLY+, SER^LOW^GLY- medium and statistical analysis. SER+/GLY+: serine 30mg/L, glycine 10mg/L, SER^LOW^GLY-: serine 10mg/L, no glycine, co-culture duration was 24 h. (**F-G**) Detection of PD-1 expression level in CD8^+^ T cells co-cultured with SERINC2 overexpressed/knockdown HeLa/C-4I cell lines in SER+/GLY+, SER^LOW^GLY- medium and statistical analysis. SER+/GLY+: serine 30mg/L, glycine 10mg/L, SER^LOW^GLY-: serine 10mg/L, no glycine, co-culture duration was 24 h **(H)** CD8^+^ T cells dead staining after 72h co-culture in complete medium with SERINC2 overexpressing HeLa/C-4I cell lines. *P < 0.05, **P < 0.01, ***P < 0.001.

**Figure 6:**
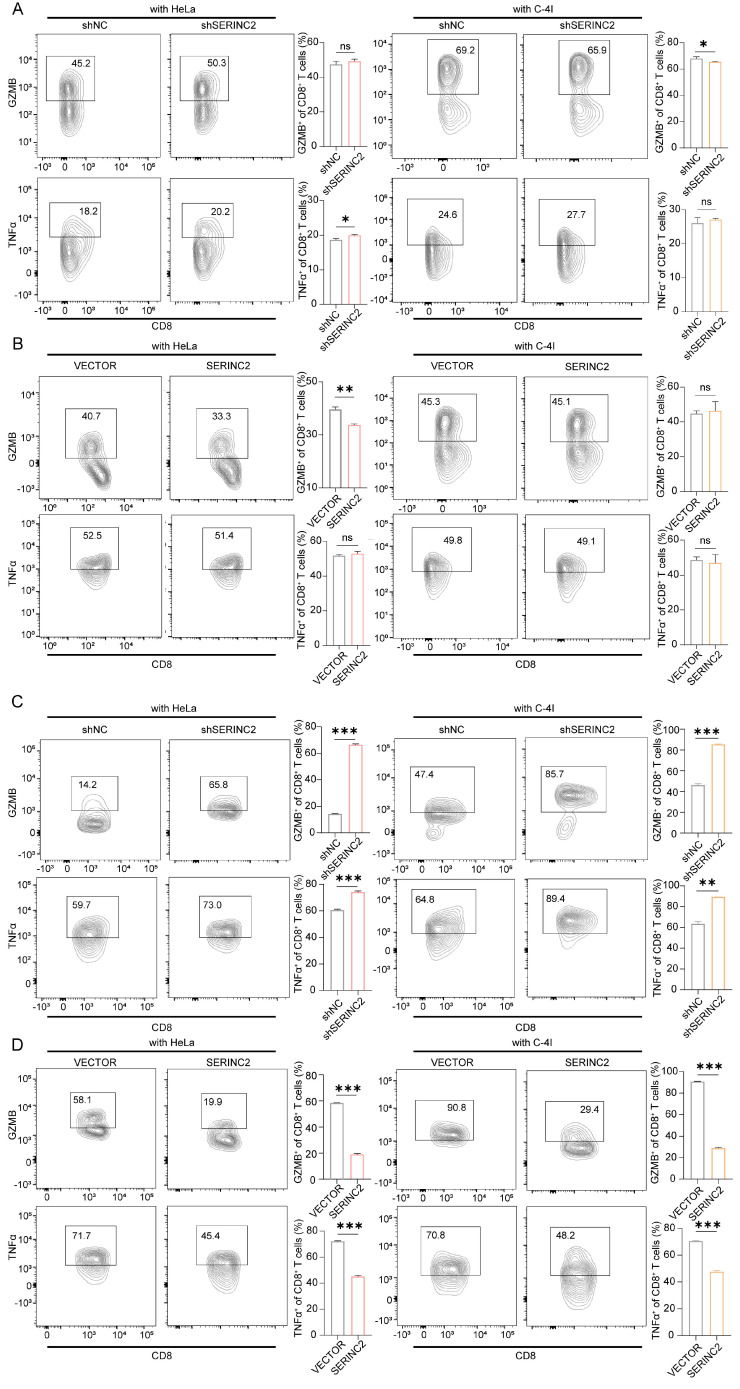
SERINC2 drives CD8^+^ T cells exhaustion in serine limited environment. **(A-B)** Detection of GZMB and TNFα expression level in CD8^+^ T cells co-cultured with SERINC2 control/knockdown, SERINC2 control/overexpressing HeLa/C-4I cell lines in normal medium and statistical analysis, co-culture duration 48h.** (C-D)** Detection of GZMB and TNFα expression in CD8^+^ T cells co-cultured with SERINC2 control/knockdown, SERINC2 control/overexpressing HeLa/C-4I cell lines in SER^LOW^GLY- medium and statistical analysis, co-culture duration was 24h. ns: not significant, *P < 0.05, **P < 0.01, ***P < 0.001.

To investigate how SERINC2 modulated the microenvironment, we created an *in vitro* TIME model by co-culturing CESC cells with human CD8^+^ T cells (Fig. 5A). As the above-mentioned results have suggested the positive regulation of SERINC2 and intracellular serine level, we hypothesized that SERINC2 probably compete for serine in TIME prior to CD8^+^ T cells. Since T cells expressed SERINC2 as well, we initially analyzed online single cell RNA sequencing database to rule out any significant SERINC2 expression by T cells over CESC cells. Analysis of the results from GSE168652 revealed the highest SERINC2 expression in malignant cells compared to adjacent normal cells and CD8^+^ T cells (Fig. 5B). In order to trace the amount of serine, we replaced the natural serine with 10mg/L of 1-13C labelled L-serine in our co-culture system to simulate the nutrient-sparse environment. Since the intracellular serine steady state reaching time was 15 min for effector T cells and plasma serine elimination half-life was1.85 to 14.81h^7^, ^37^, we then co-cultured SERINC2 overexpressed/Vector C-4I cells and CD8^+^ T cells for 4h before evaluation. Surprisingly, the amount of serine was higher in SERINC2 overexpressed C-4I cells and in its supernatant compared to the control group even with a cell number ratio of 5 to 1(CD8^+^ T cells to C-4I cells) (Fig. 5C, Supplementary Fig. 3C). This finding strongly supported the hypothesis that SERINC2 facilitates CESC cells direct or indirect serine uptake in the tumor microenvironment. In light of these findings, we investigated the impact of serine deprivation in the TIME on CD8^+^ T cells. We concurrently examined the expression of immunosuppressive markers, CTLA-4 and PD-1, on T cells in serine-abundant and serine-limited co-culture systems. The results revealed that higher SERINC2 expression correlated with elevated CTLA-4 and PD-1 expression on T cells, especially in a serine limited co-culture system with both HeLa and C-4I cells (Fig. 5D-G). In addition, the overexpression of SERINC2 induced CD8^+^ T cells death (Fig. 5H). Assessment of cytotoxic markers, GZMB and TNFα, in CD8^+^ T cells showed no significant differences in the serine-abundant co-culture system (Fig. 6A-B). However, their expressions were substantially decreased when co-cultured with SERINC2 force-expressed CESC cells in serine-limited medium (Fig. 6D). Consistently, SERINC2 knockdown restored cytotoxic function of CD8^+^ T cells (Fig. 6C). In conclusion, SERINC2 in CESC cells deprives micro-environmental serine preferentially, leading to CD8^+^ T cell exhaustion and dysfunction.

### SERINC2 regulates cervical cancer tumor immune microenvironment *in vivo*

**Figure 7:**
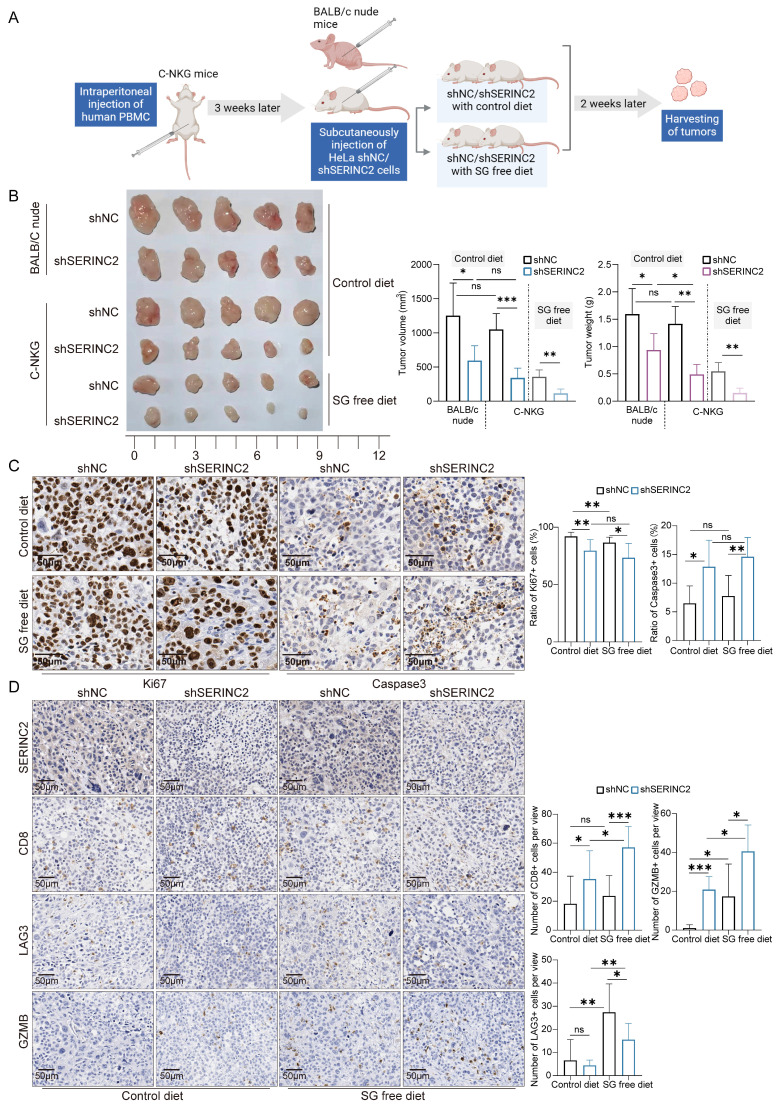
SERINC2 drives CD8^+^ T cell exhaustion *in vivo*. **(A)** Diagram of C-NKG immune establishment and xenograft assay procedure. SG free: serine and glycine free diet. **(B)** Tumorigenesis of shNC/shSERINC2 HeLa cells in BALB/C nude mice and C-NKG mice. Tumor volume and weight comparison of terminal tumors, scale bar=1 cm.** (C)** Ki67 and Caspase3 staining of subcutaneous tumor sections from C-NKG mice and statistical analysis, scale bar=50μm. **(D)** SERINC2, CD8, LAG3 and GZMB IHC staining of tumor sections from C-NKG mice and statistical analysis, scale bar=50μm.ns, not significant, *P < 0.05, **P < 0.01, ***P < 0.001.

To delve deeper into the role of SERINC2 in the TIME, we utilized C-NKG mice grafted with human immune cells and cervical cancer cells to establish *in vivo* model. Together with the C-NKG model, the BALB/c nude mice were also applied and subcutaneously injected with HeLa cells. A subgroup of C-NKG mice were fed with serine/glycine (SG) free diet while the remaining were given control diet (Fig. 7A). As a result, both serine and SERINC2 were essential for the cell growth *in vivo* (Fig. 7B). Tumor volume and weight were slightly lower but not significant in C-NKG control diet group compared to BALB/c nude mice group. In accordance with the findings *in vitro*, SERINC2 knockdown impaired HeLa cells' dependency on serine while the downregulation of SERINC2 didn't affect cell proliferation and apoptosis between SG free and control diet group (Fig. 7C). More interestingly, knockdown of either SERINC2 alone or SERINC2 plus serine could increase CD8^+^ T cells infiltration. Notably, the cytotoxic effect of T cells was significantly enhanced by downregulating SERINC2, even in a serine-limited environment (Fig. 7D). Furthermore, SERINC2 significantly augmented the expression of immunosuppressive markers LAG3, especially in SG free diet group. All these evidences indicate the pivotal role of SERINC2 in TIME regulation and progression of cervical cancer.

### Downregulation of SERINC2 impedes critical biological processes of CESC cells

**Figure 8:**
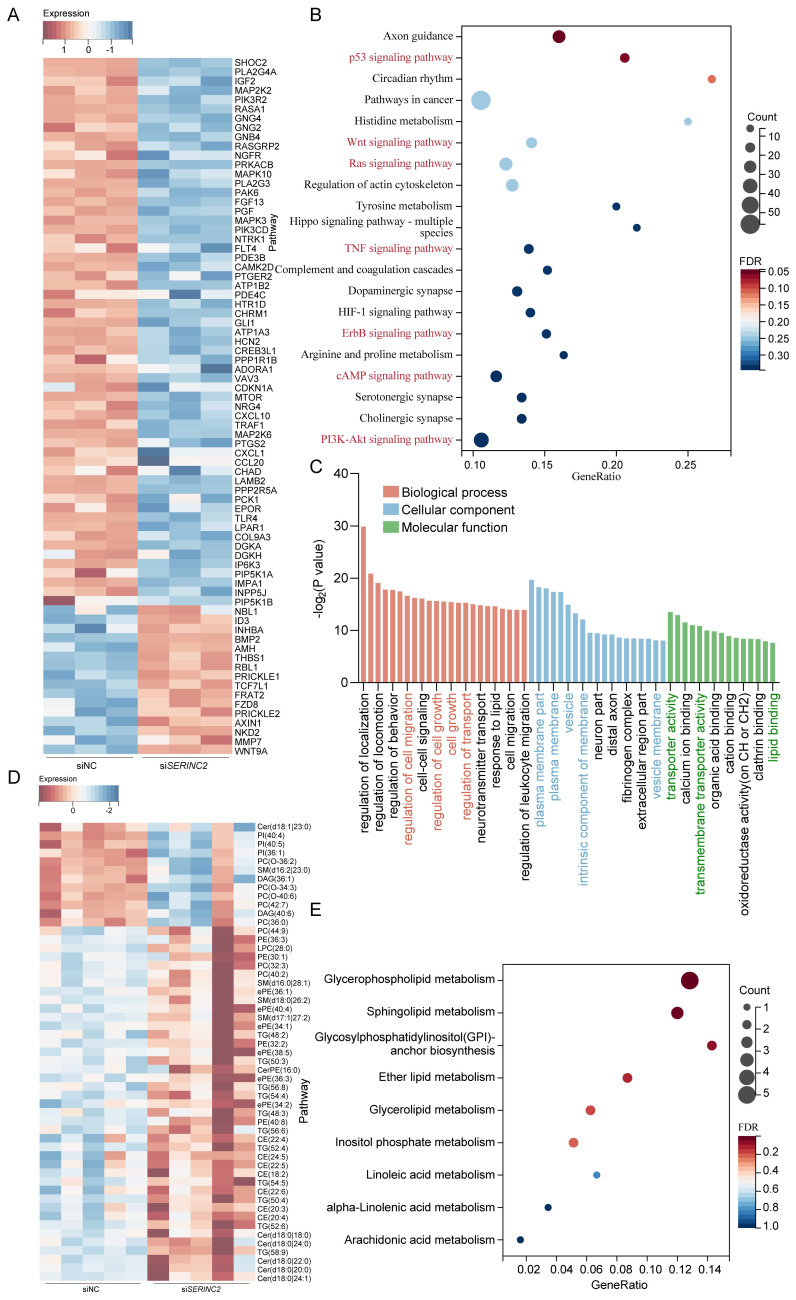
Knockdown of SERINC2 impedes CESC cell biological process. **(A)** Heatmap of representative DEGs in HeLa siNC and si*SERINC2* mRNA sequencing. **(B)** Bubble plot showing the KEGG pathway enrichment analysis of mRNA sequencing DEGs. **(C)** Top significant enrichment pathway by GO analysis of DEGs. **(D)** Heatmap of differential metabolites in HeLa siNC/si*SERINC2* lipid-targeted metabolomic study. **(E)** Bubble plot showing the KEGG pathway enrichment analysis of differential metabolites.

Subsequently, we performed mRNA sequencing in siNC/si*SERINC2* HeLa cells. Among all the DEGs, we have observed many genes that correlated with cell growth and migration (Fig. 8A). Concurrent KEGG enrichment analysis showed that several critical signaling pathways were significantly altered, suggesting the downregulation of SERINC2 modulated certain cell biological processes (Fig. 8B). Besides, gene ontology (GO) analysis indicated that SERINC2 functioned in transmembrane transport and regulation of plasma membrane (Fig. 8C). According to the previous results, SERINC2 did not mainly exert its function in cytosol. We deduced that in cervical cancer, SERINC2 also played its role on membrane, regulating serine derived lipid such as sphingolipid and phosphatidylserine. To verify this hypothesis, a lipid-targeted metabolomic study was conducted on siNC/si*SERINC2* HeLa cells. Along with the downregulation of SERINC2, phosphatidylcholine (PC (O-34:3), PC (O-36:2), PC (42:7), PC (36:0)) and sphingolipid (ceramide (Cer d18:1|23:0), sphingomyelin (SM d16:2|23:0)) has significantly decreased (Fig. 8D). In the meantime, pathway enrichment analysis indicated that SERINC2 regulated the membrane-component-associated metabolism including glycerophospholipid metabolism, sphingolipid metabolism and glycosylphosphatidylinositol (GPI)-anchor biosynthesis. Taken together, SERINC2 might play an important role in facilitating transporting of serine and regulating membrane associated process, renders the alteration of intracellular signaling.

## Discussion

In this study, we uncovered that SERINC2 was a critical serine-associated gene and significantly upregulated in cervical cancer. Importantly, the expression of SERINC2 predicted poor prognosis in CESC patients. We found that CESC cells relied on serine for proliferation and it was in a SERINC2-dependent mode. SERINC2 promoted the progression of cervical cancer *in vitro* and *in vivo*. On the other side, SERINC2 expressed in CESC cells competed for serine preferentially in TIME, rendered dysfunction and exhaustion of CD8^+^ T cells. More importantly, SERINC2 located to cell membrane and modulated membrane associated metabolism, thus regulating several critical signaling pathways. All in all, the understanding of SERINC2 may provide us with a new approach in cervical cancer immunotherapy.

Our knowing about SERINC2 is still limited. Previously, SERINC2 was more reported in nervous system diseases such as bipolar disorder, autism spectrum disorder and alcohol dependence^38^-^40^. In addition to that, SERINC2 was also investigated in immune. A study using SERINC2 knockout acute lung injury (ALI) mouse model has unveiled that Serinc2-KO mice exhibit exacerbated ALI-related pathologies after cecal ligation and puncture. The expressions of pro-inflammatory factors such as IL1β, IL6, TNFα, and MCP1 were significantly enhanced by Serinc2 deficiency. Besides, Serinc2 overexpression in RAW264.7 cells significantly suppressed the lipopolysaccharide-induced inflammatory response^41^. Another study in intervertebral disc degeneration identified *SERINC2* as key characteristic gene because it was related to the proportions of T cells gamma delta and neutrophils^42^. However, the real mechanism of SERINC2 remains unclear. Initially, we were convinced that SERINC2 related to phosphatidylserine biosynthesis in cervical cancer cells. Nevertheless, more metabolomic studies are required to support this presumption. In RNA sequencing results, we have observed that several phospholipid scramblase protein coding gene decreased too upon with downregulation of SERINC2. We guessed SERINC2 was also likely to modulate the flipping in and out activity of PS. In any case, it will be another story but irrelevant to this research.

Studies have provided mechanistic details of function of sphingolipids in tumor growth regulation^43^, ^44^. For example, the CERS1generated C18 ceramide induced cell death and tumor suppression in head and neck squamous cell carcinoma^45^. Another study has demonstrated an increase in C16 ceramide level in breast cancer tissue compared to normal tissue^46^. So far, many cancer drugs targeting sphingolipids metabolism have been put into clinical trials^47^. Our study has indicated the association of SERINC2 and sphingolipid metabolism, which can be deemed as a promising target in cervical cancer therapy. As SERINC2 is a transmembrane protein, a targeted antibody could be designed to specifically aim at SERINC2. The loss of SERINC2 would impair cell viability and remodel TME, releasing more nutrients including serine and benefiting the proliferation of cytotoxic T cells.

In this research we have discussed the impact of SERINC2 on CD8^+^ T cells. We applied CTLA-4 rather than PD-1 as principal marker to represent exhaustion. However, it was reported that the expression of PD-1 also represented the anti-tumor reactivity^48^. As a matter of fact, the exhausted T cells were comprised of multiple transcriptionally and epigenetically distinct populations^49^. A subset of them still exhibited a robust effector phenotype. Hence, further research needs to be done to determine the characteristics of exhausted CD8^+^ T cells in this study. Besides, this research has other limitations. We have discovered that SERINC2 increased serine intake and modulate lipid metabolism in cervical cancer. Yet, we are still unaware how SERINC2 assists the transport and utilization of serine. More evidences are needed to fill in our knowing about SERINC2. The isotope labeling and metabolic flux are required for the further investigation.

In conclusion, our findings indicate that targeting SERINC2 is a promising therapeutic target in CESC immunotherapy. Functional exploration of SERINC2-mediated serine metabolism and nutrients competition unveil the mechanism of cervical cancer progression and T cell exhaustion.

## Supplementary Material

Supplementary figures and tables.
