# Supplementary material for: SERINC2-mediated serine metabolism promotes cervical cancer progression and drives T cell exhaustion
Source: Int J Biol Sci. 2025 Jan 20;21(3):1361–77. doi: 10.7150/ijbs.105572 (PMC11781177; doi:10.7150/ijbs.105572)
Supplement: Supplementary file 1 — Supplementary figures and tables. [file ijbsv21p1361s1.pdf]

SERINC2-mediated serine metabolism promotes cervical cancer progression and drives T cell exhaustion

Yixuan Sun1*, Yang Zhou1*, Qihua Peng1*, Wanzhen Zhou1*, Xiao Li1, Ruiwen Wang1, Yifan Yin3, Huixian Huang1, Hongfei Yao4, Qing Li2, Xueli Zhang2, Lipeng Hu2, Shuheng Jiang2, Zhigang Zhang2, Dongxue Li2[#], Xiaolu Zhu1[#], Yincheng Teng1[#]

1. Department of Gynecology and Obstetrics, Shanghai Sixth People's Hospital Affiliated to Shanghai Jiao Tong University School of Medicine, Shanghai, 200233, P.R. China.
2. State Key Laboratory of Systems Medicine for Cancer, Shanghai Cancer Institute, Ren Ji Hospital, School of Medicine, Shanghai Jiao Tong University, Shanghai, 200240, P.R. China.
3. Department of Biliary-Pancreatic Surgery, Ren Ji Hospital, School of Medicine, Shanghai Jiao Tong University, Shanghai, 200127, P.R. China.
4. Department of General Surgery, Pancreatobiliary Surgery Center, Huadong Hospital Affiliated to Fudan University, Shanghai, 200040, PR China.

#Corresponding authors: Yincheng Teng, Shanghai e-mail address: teng_yc@126.com
ORCID:0000-0002-6451-4479
Xiaolu Zhu, Shanghai e-mail address: zhuxl_ong@163.com
Dongxue Li, Shanghai e-mail address: dxli@shsci.org

*Yixuan Sun, Yang Zhou, Qihua Peng and Wanzhen Zhou contributed equally to this research

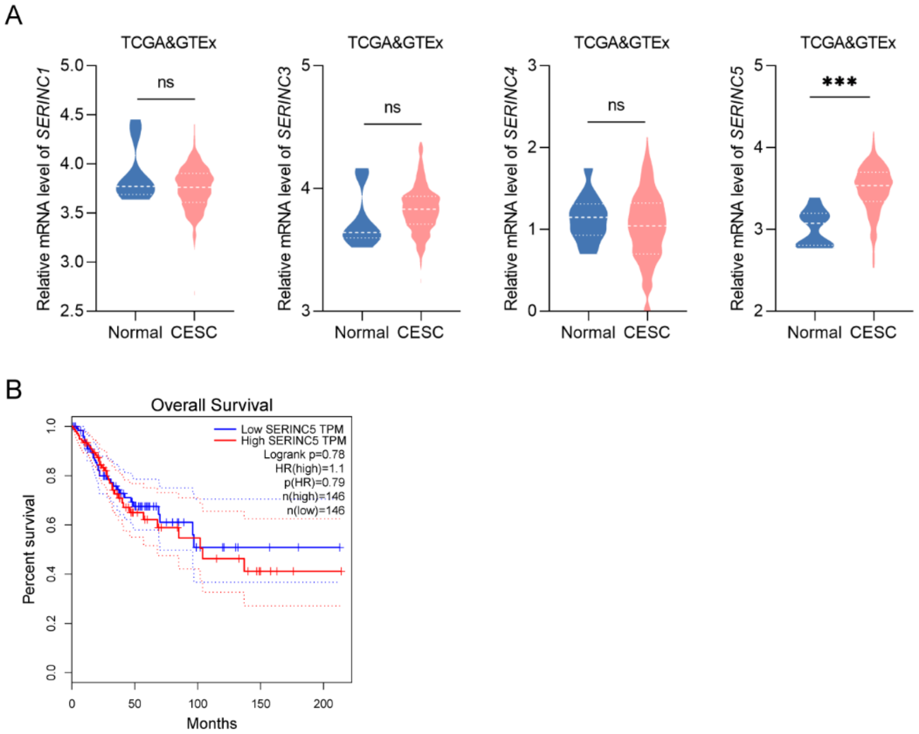

Supplementary Fig.1 Expression profile and prognosis analysis of SERINC family genes. **(A)** Expression of *SERINC1*, *SERINC3*, *SERINC4* and *SERINC5* using TCGA and GTEx database. **(B)** Kaplan-Meier analysis of OS in patients with high or low *SERINC5* expression using GEPIA online server. ns: not significant, $*P < 0.05$, $**P < 0.01$, $***P < 0.001$

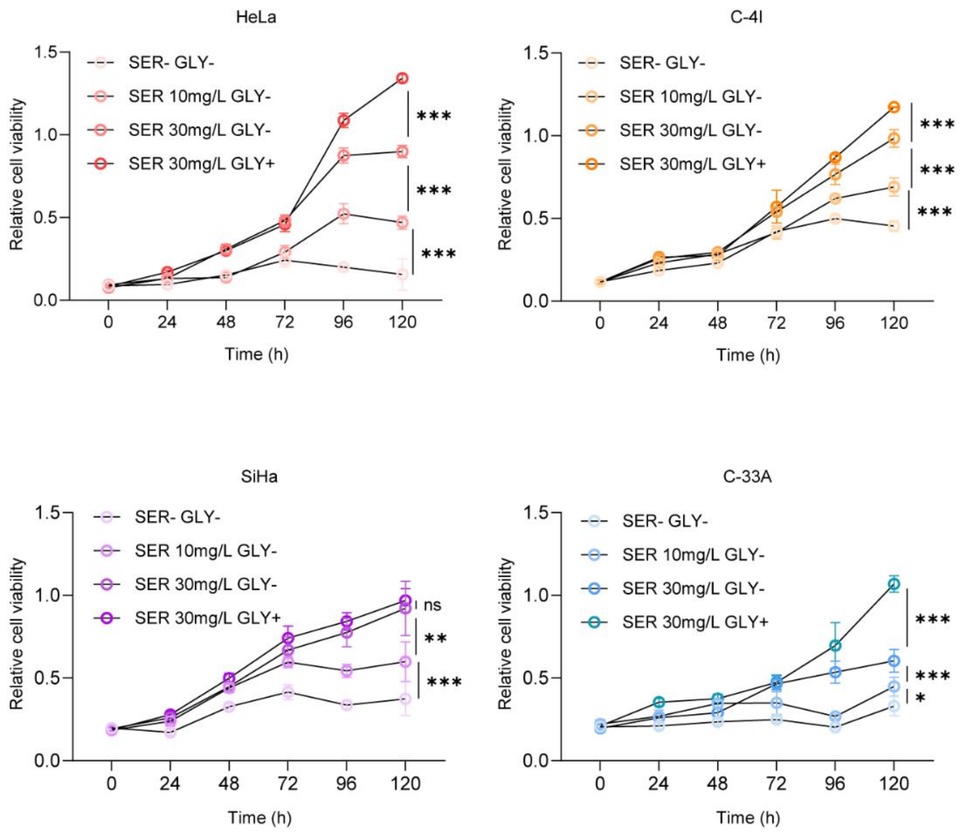

Supplementary Fig.2 The growth of cervical cancer cells depended on serine. CCK-8 analysis of HeLa, C-4I, C-33A and SiHa cell viability under different serine concentration (serine 0mg/L, 10mg/L, 30mg/L, without glycine and serine 30mg/L with glycine 10mg/L) with the analysis. **P* < 0.05, ***P* < 0.01, ****P* < 0.001

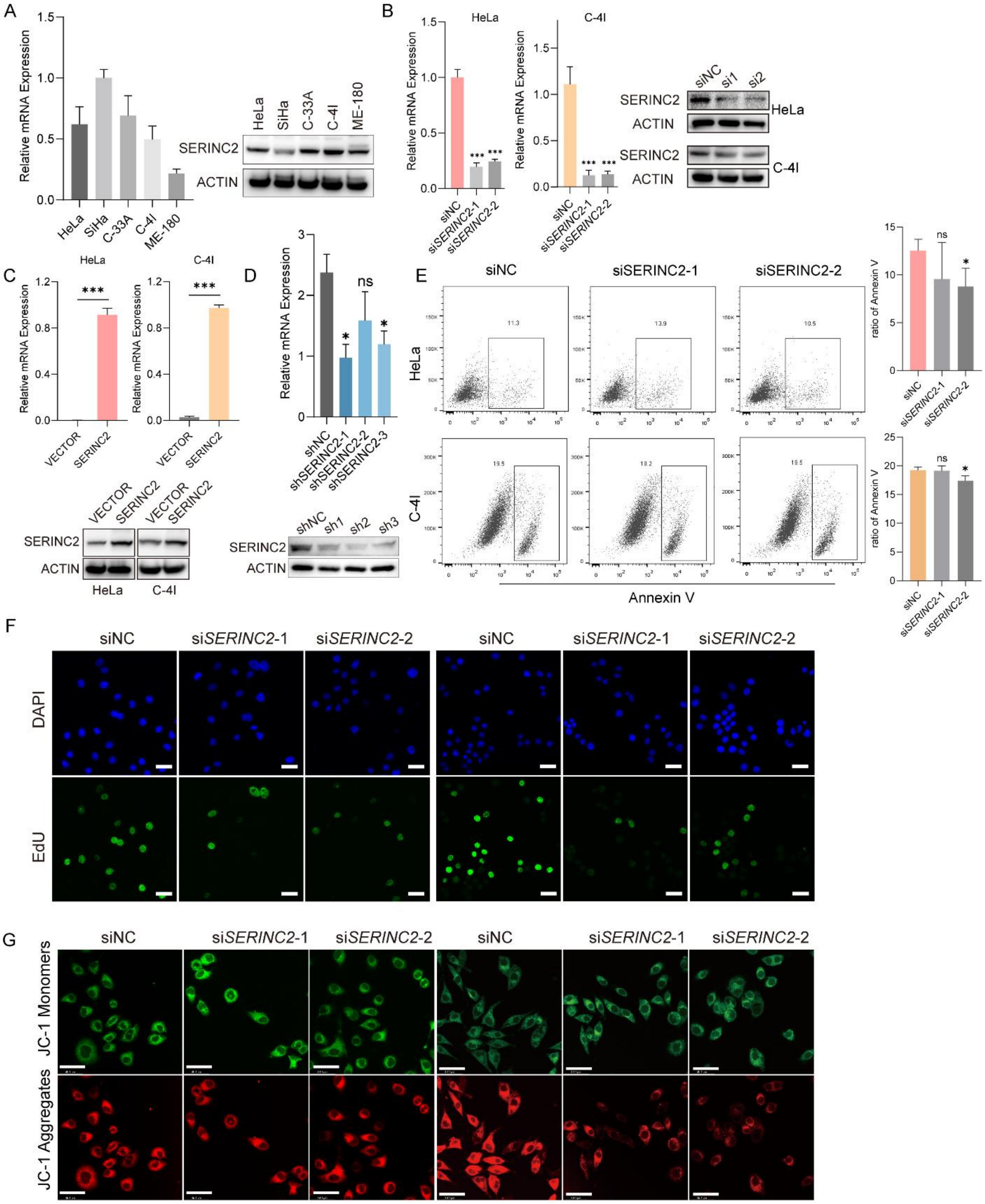
A
Relative mRNA Expression
HeLa
SiHa
C-33A
C-4I
ME-180
SERINC2
ACTIN
B
HeLa
C-4I
Relative mRNA Expression
siNC
siSERINC2-1
siSERINC2-2
siNC
si1
si2
SERINC2
ACTIN
C
VECTOR
SERINC2
D
shNC
shSERINC2-1
shSERINC2-2
shSERINC2-3
ns
sh1
sh2
sh3
E
siNC
siSERINC2-1
siSERINC2-2
Annexin V
ratio of Annexin V
F
DAPI
EdU
G
JC-1 Monomers
JC-1 Aggregates

Supplementary Fig.3 SERINC2 cell line expression, knockdown/overexpressed efficiency and functional assay. **(A)** SERINC2 expression in HeLa, C-4I, C-33A, SiHa and ME-180 cells at mRNA and protein level. **(B)** Validation of SERINC2 knockdown efficiency at mRNA and protein level in HeLa and C-4I cells. **(C)** Validation of SERINC2 overexpression efficiency at mRNA and protein level in HeLa and C-4I cells. **(D)** Validation and statistical analysis of knockdown efficiency at mRNA and protein expression level of shNC/shSERINC2 HeLa cells. **(E)** Apoptosis assay staining the effect of siNC/si*SERINC2* HeLa and C-4I cells with analysis. **(F)** Representative EdU staining of SERINC2 knockdown HeLa and C-4I cells in separate channel. Scale bar = 50μm. **(G)** Representative JC-1 staining images of SERINC2 knockdown HeLa and C-4I cells in separate channel. Scale bar = 50μm.*$P < 0.05$, **$P < 0.01$, ***$P < 0.001$

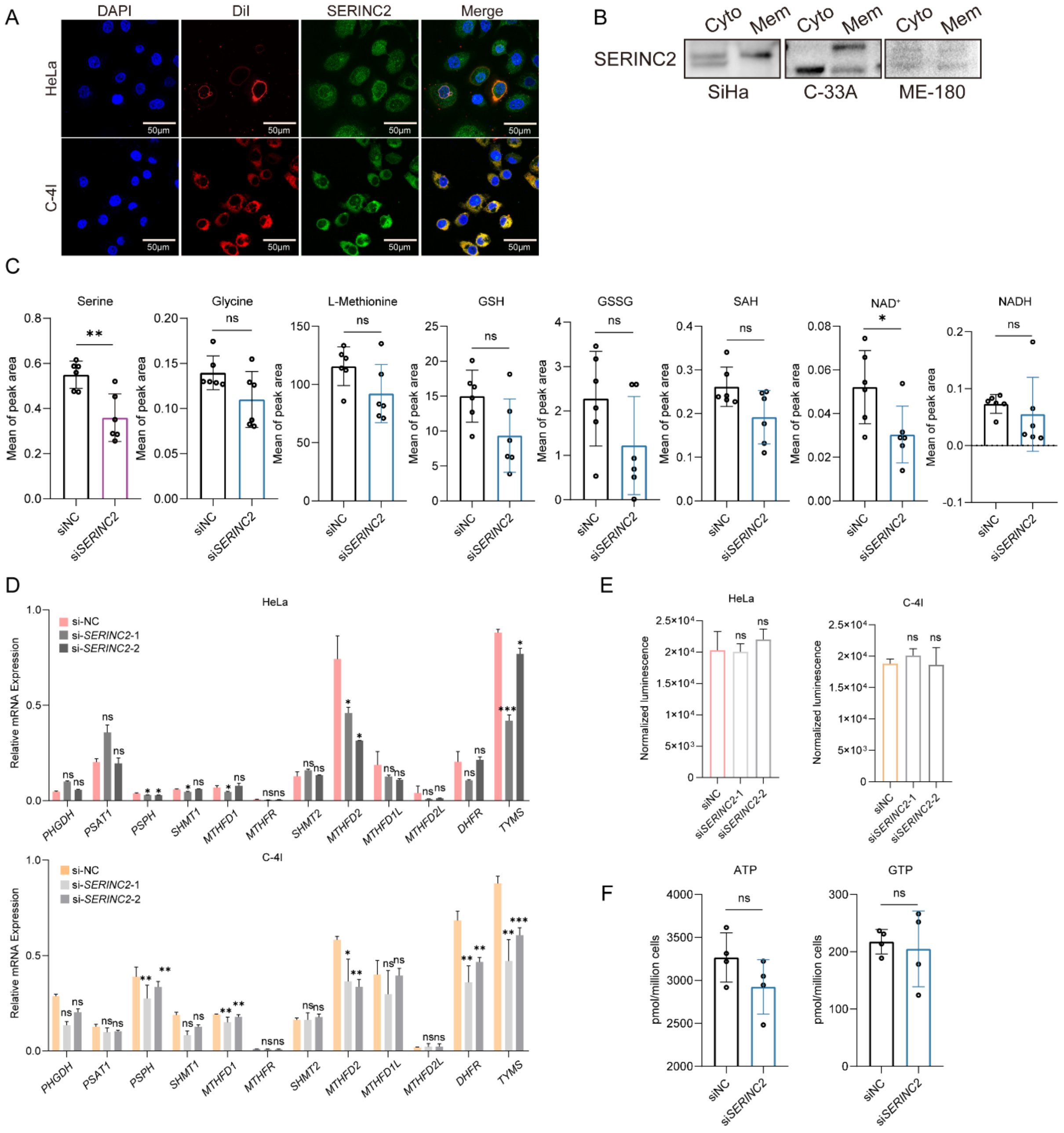

Supplementary Fig.4 SERINC2 didn't involve in SGOC metabolism. **(A)** IF staining of SERINC2 expression and localization in HeLa and C-4I cells. DiI: lipophilic carbocyanine fluorescent dye used to target cell membrane. scale bar: 50 μm. **(B)** Western blotting using protein extracts from cytoplasm and membrane to determine SERINC2 expression in SiHa, C-33A and ME-180 cells. **(C)** Western blotting was applied to detect change in the AKT-mTOR pathway after SERINC2 knockdown. Puromycin infiltration assay was used to detect the synthesis of nascent protein after SERINC2 knockdown. **(D)**

mRNA protein level expression of ATF in SERINC2 knockdown HeLa, C-4I cells under normal complete medium conditions and serine/glycine deprived cultured HeLa and C-4I cells. The serine withdrawal time was 24h. **(E)** Relative quantification of serine derived SGOC metabolites after knockdown of SERINC2 using LC-MS. **(F)** mRNA level of SGOC metabolism enzyme after SERINC2 knockdown. **(G)** Intracellular ATP levels of SERINC2-knockdown HeLa and C-4I cells by ATP assay kit. **(H)** Determination of intracellular ATP and GTP level using LC-MS after SERINC downregulation in HeLa cells. ns: not significant, *$P < 0.05$, **$P < 0.01$, ***$P < 0.001$

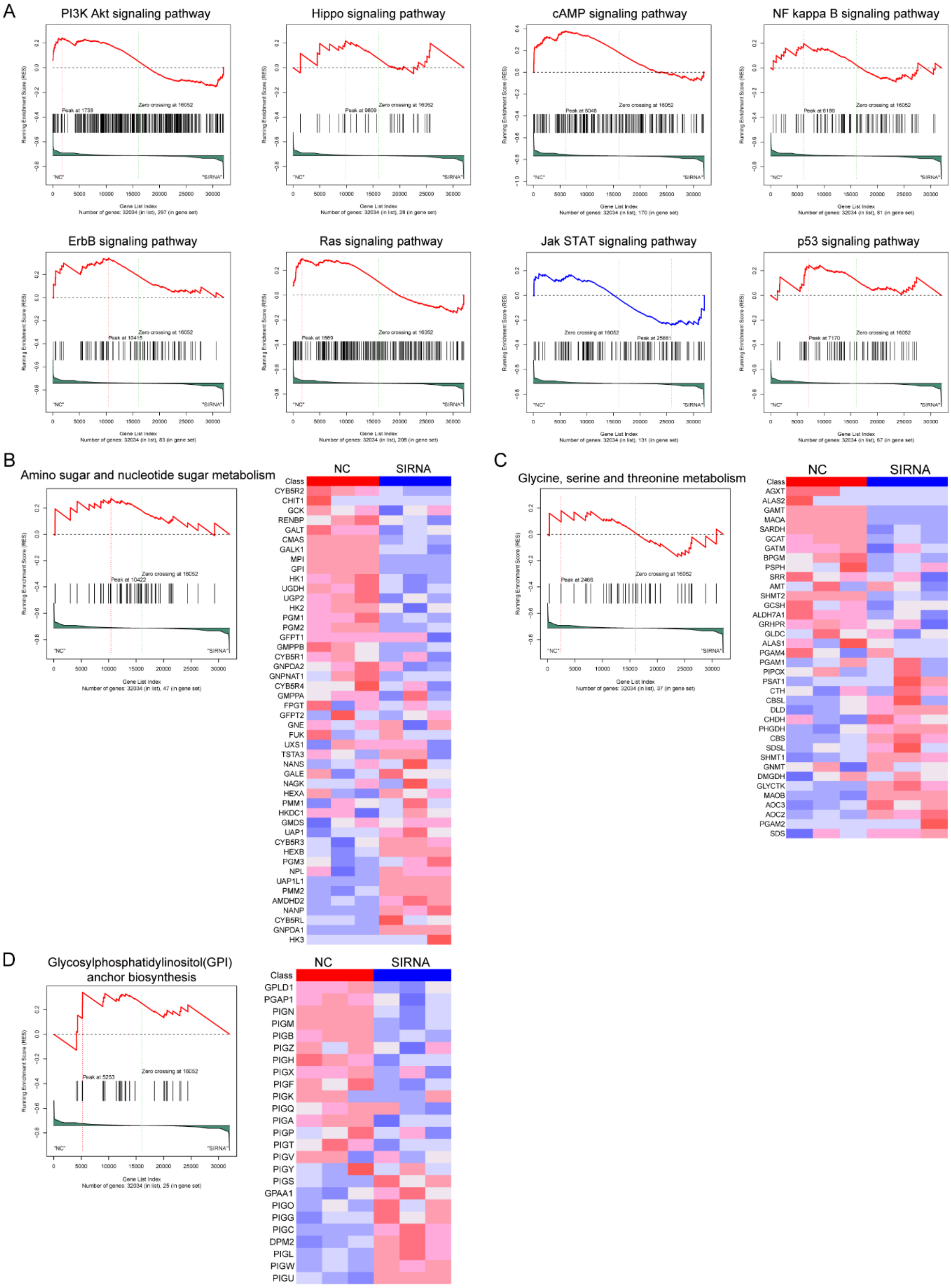

Supplementary Fig.5 Pathway enrichment analysis of SERINC2-knockdown HeLa cells. **(A)** GSEA analysis revealed several critical pathways that enriched in si*SERINC2* HeLa cells compared to siNC HeLa cells. **(B-D)** GSEA analysis and gene expression profiles in si*SERINC2* HeLa cells versus siNC

HeLa cells

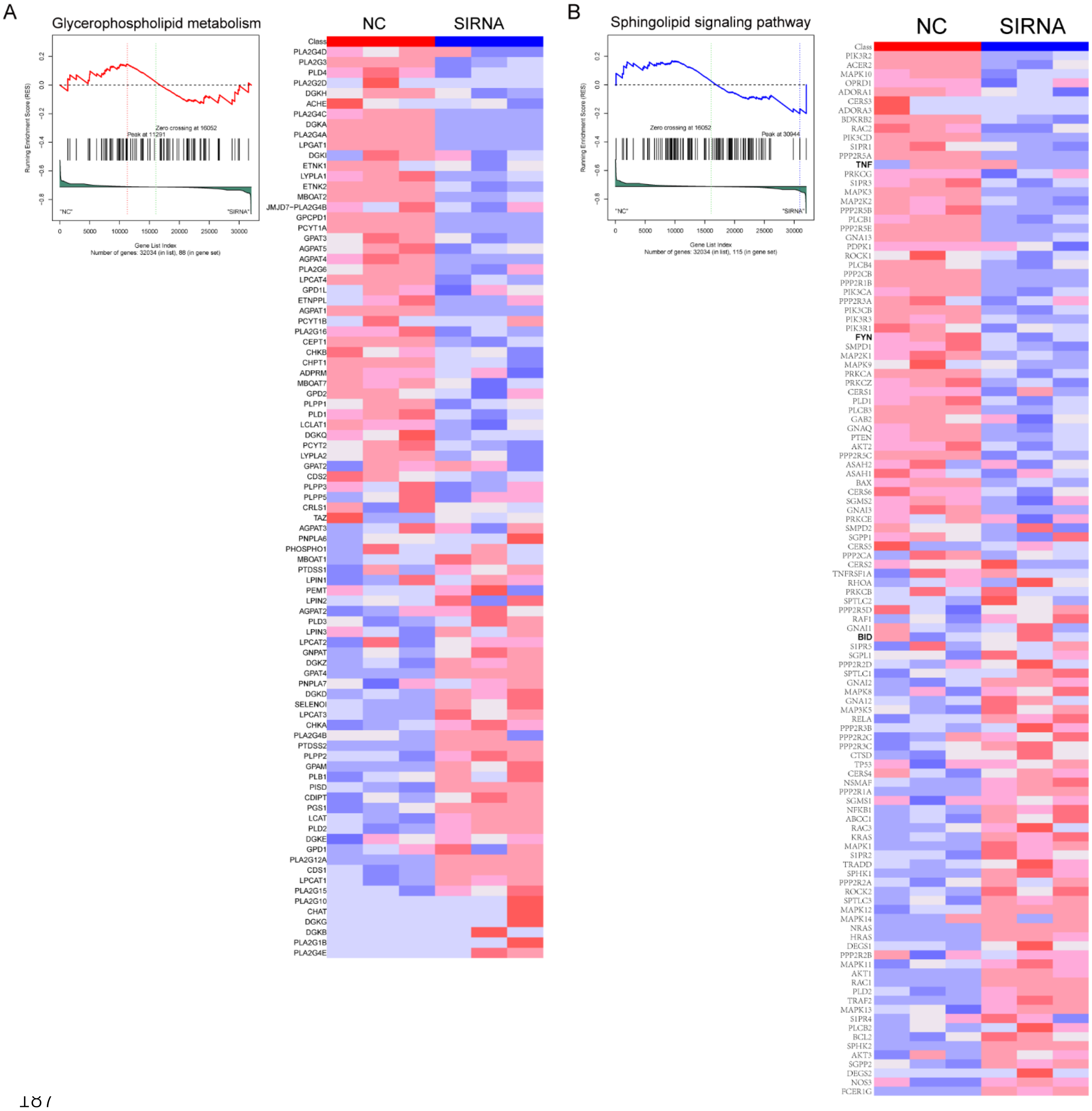

Supplementary Fig.6 Pathway enrichment analysis of SERINC2-knockdown HeLa cells. **(A, B)** GSEA analysis and gene expression profiles in si*SERINC2* HeLa cells versus siNC HeLa cells.

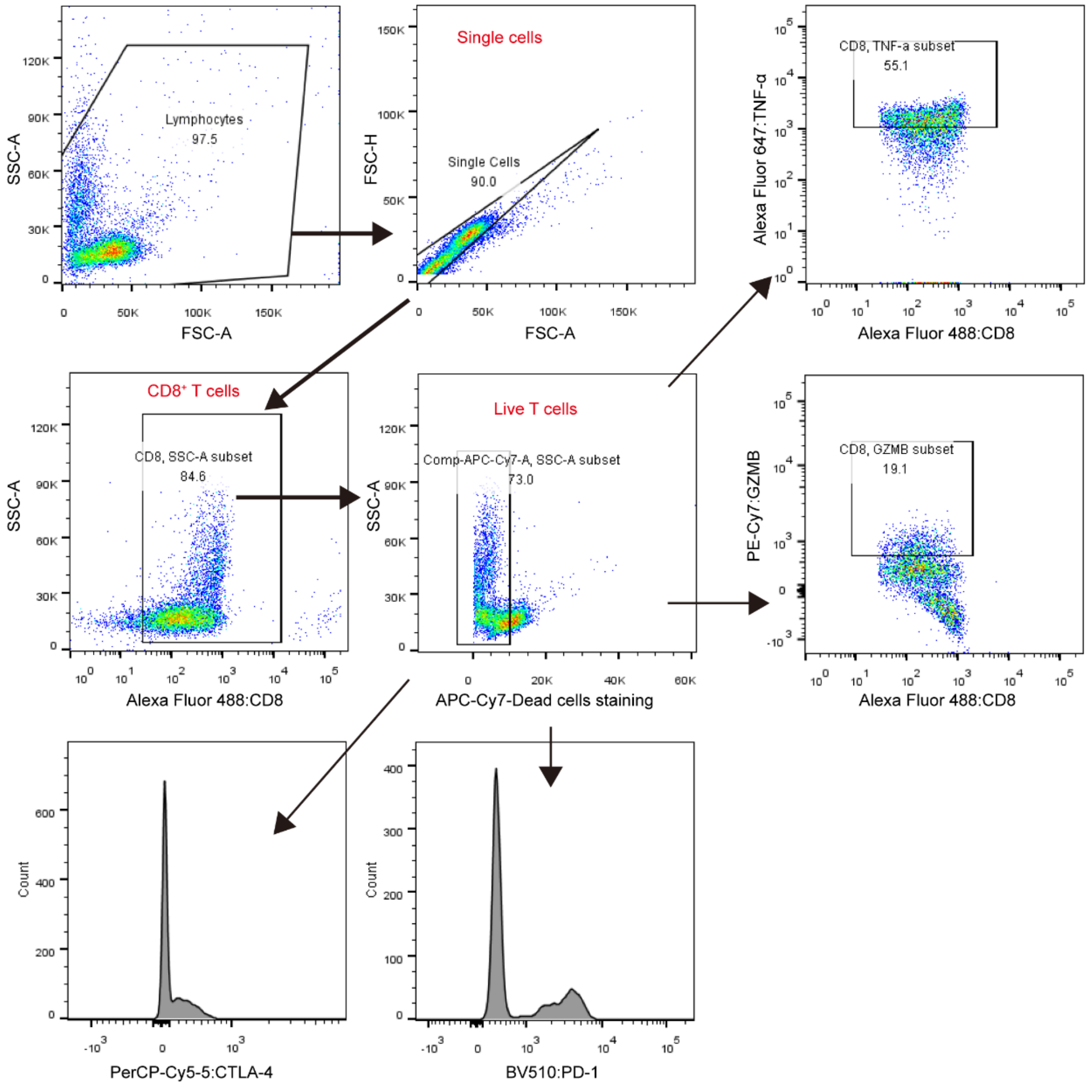

Supplementary Fig.7 Gating strategy for CD8$^+$ T cells functional assay.

Supplementary Table1 Primers used in this study.

	Forward Primer (5'-3')	Reverse Primer (5'-3')
ATF4	TCCAACAACAGCAAGGAGGA	TACCCAACAGGGCATCCAAG
PHGDH	CTGCGGAAAGTGCTCATCAGT	TGGCAGAGCGAACAATAAGGC
PSAT1	TGCCGCACTCAGTGTTGTTAG	GCAATTCCCGCACAAGATTCT
PSPH	GAGGACGCGGTGTCAGAAAT	GGTTGCTCTGCTATGAGTCTCT
SHMT1	CTGGCACAACCCCTCAAAGA	AGGCAATCAGCTCCAATCCAA
MTHFD1	AGGATGTGGATGGATTGACTAGC	CCCTTAGGCGTACAAGGAATG
MTHFR	CCGCCGTGAACTACTGTGG	AGATGGCCCGTGATCTCCTC
SHMT2	CATCGTCACCACCACTACTCACAAG	CAGGGATGGGAACACGGCAAAG
MTHFD2	GATCCTGGTTGGCGAGAATCC	TCTGGAAGAGGCAACTGAACA
MTHFD1L	GGCTCTGTATAATCGGCTGGTTCC	CTCTTCTGTCAGTGTGCTCGGATC
MTHFD2L	CAGTTACCACTACCAGACCACG	GGCAGGTATGAGAGAATGCTGA
DHFR	GAGAACTCAAGGAACCTCCACAAGG	CAGAACTGCCACCAACTATCCAGAC
TYMS	GGAGTGAAAATCTGGGATGCC	ACTGGAAGCCATAAACTGGGC

Supplementary Table 2 Antibody used in this study.

	Catalogue Number	Company
Anti-mTOR	2972S	Cell Signaling Technology
Anti-p-mTOR(S2481)	2974T	Cell Signaling Technology
Anti-AKT	9272S	Cell Signaling Technology

Anti-p-AKT(S473)	9271T	Cell Signaling Technology
Anti-Puromycin	MABE343	Merck
Anti-ATF4	10835-1-AP	Proteintech
Anti-Na,K-ATPase	GB11400-100	Servicebio

Cell membrane and cytosol protein extraction assay

Cell membrane and cytosol protein extraction was performed using Membrane and Cytosol Protein Extraction Kit (Beyotime, China, Cat#P0033) according to manufacturer's guideline. Briefly, a total of $1x10^7$ cells were harvested and resuspended in reagent A containing phenylmethanesulfonyl fluoride (PMSF, Beyotime, China, Cat#ST506). The samples were subjected to 2 cycles of freeze and thaw by liquid nitrogen, followed by centrifugation at 700 g for 10 mins in 4 ℃. The supernatant was then centrifuged at 14000 g for 30 mins and the precipitation was resuspended in reagent B. After ice-bath for 40 mins, samples were centrifuged at 14000 g for 5 mins and the supernatant was maintained at -80℃ or prepared for immunoblotting sample loading.
